# Incidence of traumatic cervical spine fractures in the Norwegian population: a national registry study

**DOI:** 10.1186/s13049-014-0078-7

**Published:** 2014-12-18

**Authors:** Hege L Fredø, Inger J Bakken, Bjarne Lied, Pål Rønning, Eirik Helseth

**Affiliations:** Department of Neurosurgery, Oslo University Hospital, Oslo, Norway; Faculty of Medicine, University of Oslo, Oslo, Norway; Norwegian Patient Register, the Norwegian Directorate of Health, Trondheim, Norway; Present address: The Norwegian Institute of Public Health, Oslo, Norway

**Keywords:** Cervical vertebrae, Spinal fractures, Trauma, Spinal cord injuries, Incidence, Epidemiology

## Abstract

**Background:**

The incidence of cervical spine fractures (CS-fx) in the general population is sparingly assessed. The aim of the current study was to estimate the incidence of traumatic CS-fx and of open surgery of cervical spine injuries in the Norwegian population.

**Methods:**

The Norwegian Patient Register (NPR) is an administrative database that contains activity data from all Norwegian government-owned hospitals and outpatient clinics. The diagnoses and procedures are coded according to the International Statistical Classification of Diseases and Related Health Problems (ICD-10) and the NOMESCO Classification of Surgical Procedures (NCSP), respectively. We retrieved information on all severe traumatic cervical spine injuries between 2009 and 2012 from the NPR. Updated information on the date of death is included through routine linkage to the General Register Office.

**Results:**

Between 2009 and 2012, a total of 3 248 patients met our criteria for severe traumatic cervical spine injury. A total of 2 963 patients had one or more CS-fx, and 285 had severe non-fracture cervical spine injuries. The median age was 54 years, and 69% of the patients were male. The incidence of CS-fx and severe non-fracture injuries in the total Norwegian population was 16.5/100 000/year, and the incidence of CS-fx was 15.0/100 000/year. A total of 18% of the patients were treated with open surgery, resulting in an estimated incidence of surgery for acute traumatic cervical spine injury of 3.0/100 000/ year in the Norwegian population. The 1- and 3-month mortality rates were 4% and 6%, respectively.

## Background

The incidence of traumatic cervical spine fractures (CS-fx) in a general population has so far only been reported from Sweden, Canada and Norway [1-3]. Several other reports have described the incidence of CS-fx, but most of these studies have involved different subpopulations, such as trauma center patients, specific age groups, head injury patients, military populations and osteoporotic patients [4-19]. The Swedish national survey of traumatic cervical injuries reported an incidence of CS-fx of 9.2/100 000/year [1]. In 1996, Hu et al. reported that the incidence of all traumatic spinal fractures in a Canadian population was 64/100 000/year [2]. Based on the proportion of hospitalized patients in the Canadian study, the incidence of CS-fx could be estimated at 12/100 000/year.

In Norway, all inpatient health care for acute traumatic conditions is performed in government-owned hospitals. Health care is organized into four health care regions based on geographic location; Southeast, West, Central and North Norway. In Southeast Norway, all CS-fx surgeries are performed at a single center. We recently estimated the incidence of CS-fx in Southeast Norway to be 11.8/100 000/year, based on prospective injury registration at this center [3]. This rate is likely an underestimate because a minor proportion of patients who are evaluated at local hospitals and have a CS-fx that does not require surgical fixation are not referred to the main trauma center for consultation or treatment.

The Norwegian Patient Register (NPR) is an administrative database containing activity data from all Norwegian government-owned hospitals and outpatient clinics. Reporting data to the NPR is mandatory, and these data are linked to the government reimbursement system for the funding of health services. The database includes a large number of clinical and non-clinical data for each hospital stay and outpatient visit.

Whereas the NPR was established in 1997, the 11-digit personal identification number unique to each individual Norwegian citizen has only been reported since 2008, after a change in the health registry act was passed by the Norwegian Parliament on March 1, 2007. Consequently, individual-level data are only available since 2008.

The aim of the current study was to estimate the incidences of CS-fx and open surgical fixation of severe traumatic cervical spine injuries in the complete Norwegian population using data from the NPR. A better understanding of the demographics of the patient population will provide the opportunity to identify persons at risk and can eventually lead to prophylactic measures.

## Methods

As of January 1, 2011, the Norwegian population was 4 920 000 (Southeast: 2 745 000; West: 1 027 000; Central: 680 000; and North: 468 000) [20].

### NPR database

Using data from the NPR, we obtained access to relevant clinical and demographic information from all government-owned hospitals and outpatient clinics in the country. Among these data are the hospitalization and discharge dates, patient demographics (sex, age and municipality), diagnostic codes (primary and secondary) and procedures and level-of-care codes (inpatient/outpatient). The diagnoses and procedures are coded according to the International Statistical Classification of Diseases and Related Health Problems (ICD-10) [21] and the NOMESCO Classification of Surgical Procedures (NCSP), respectively [22].

Updated information on the date of death is included through routine linkage to the General Register Office.

To retrieve information on all severe traumatic cervical spine injuries, including both CS-fx and severe non-fracture cervical spine injuries, we first used the criteria in Table 1 to select all traumatic CS-fx, discoligamentous (DCL) rupture and cervical spinal cord injury (cSCI) episodes that occurred from 2008 to 2012. Because DCL injury covers a broad range of conditions, DCL cases without relevant surgical procedure codes were excluded to ensure that the data set only included truly severe injuries.

**Table 1:** **List of** **ICD-10 and NCSP codes used**

	**Code**	**Text**
ICD-10	S12.0	Fracture of first cervical vertebra (atlas)
	S12.1	Fracture of second cervical vertebra (axis)
	S12.2	Fracture of other specified cervical vertebra
	S12.7	Multiple fractures of cervical spine
	S12.9	Fracture of neck, region unspecified (vertebra, spine NOS)
	S13.0*	Traumatic rupture of cervical intervertebral disc
	S13.1*	Dislocation of cervical vertebra
	S14.0	Concussion and edema of cervical spinal cord
	S14.1	Other and unspecified injury of cervical spinal cord
NCSP	ABC10	Microsurgical excision of cervical intervertebral disc displacement
	ABC21	Anterior decompression of cervical spine with insertion of interbody fixating implant
	ABC30	Decompression of cervical nerve roots
	ABC50	Decompression of cervical spinal canal and nerve roots
	ABC60	Decompression of cervical spinal cord
	NAG40/41	Interbody fusion of cervical/cervico-thoracic spine with internal fixation
	NAG70/71	Interlaminary fusion of cervical/cervico-thoracic spine with fixation
	NAJ00/01	Closed reduction of fracture of cervical/cervico-thoracic spine
	NAJ10/11	Open reduction of fracture of cervical/cervico-thoracic spine
	NAJ20/21	External fixation of fracture of cervical/cervico-thoracic spine
	NAJ30/31	Internal fixation of fracture of cervical/cervico-thoracic spine using bio-implant
	NAJ40/41	Internal fixation of fracture of cervical/cervico-thoracic spine using wire, rod, cerclage or pin
	NAJ60/61	Internal fixation of fracture of cervical/cervico-thoracic spine using plate and screws
	NAJ70/71	Internal fixation of fracture of cervical/cervico-thoracic spine using screws alone
	NAJ80/81	Internal fixation of fracture of cervical/cervico-thoracic spine using other/combined material
	NAJ90/91	Other fracture surgery of cervical/cervico-thoracic spine

*Only included if a relevant surgical procedure was also registered.

Throughout the study period, a total of 10 376 episodes were identified in 4 175 patients. Personal identifiers were available for 97.6% of these episodes. In cases lacking personal identification numbers, the NPR routinely uses the patient number unique to each patient within the hospital and the year as a substitute.

The data in the NPR do not distinguish between the first and later treatments for the same condition. To obtain incidence estimates, all of the data for patients who were first registered in 2008 (n = 927) were excluded from the data set. Thus, the study population included 3 248 patients who were registered with traumatic cervical spine injuries from 2009 to 2012. No patients were registered more than once throughout the study period.

### Patient groups

Using these criteria, the patients identified with traumatic cervical spine injuries had the following characteristics:

- one or more CS-fx or
- a severe non-fracture cervical spine injury.

Severe non-fracture cervical spine injury was defined as a DCL injury with an accompanying relevant procedure code for open surgery and/or traumatic cervical spinal cord injury (cSCI), without a coincidental CS-fx .

All of the patients were grouped according to treatment as follows:

- open surgery (surgical procedure code registered); or
- external fixation with a halo vest only (NAJ20 and/or NAJ21); or
- no open surgery or halo vest. Patients were also categorized in this group if they were registered with NAJ00/01, which is usually used for temporary skull traction, as the only procedure code.

### Ethics

The NPR is responsible for de-identifying and ensuring the anonymity of the personal data that are released in accordance with the provisions detailed in the applicable laws and regulations.

The analysis data file did not contain any information that could be traced to specific subjects. The use of such anonymous research files does not require regulatory ethics approval in Norway.

### Statistics

Statistical analyses were performed using PAWS Statistics for Windows, version 18.0 (SPSS, Inc., Chicago, Illinois, USA). The summary statistics are provided as the means ± the standard deviations or as the medians with the interquartile ranges, as appropriate. The incidence rates and their associated confidence intervals were calculated using a Poisson distribution. The age variable was created using the middle values in the age groups as a linear variable. A p-value <0.05 was considered significant.

## Results

### Patient characteristics

Between 2009 and 2012, a total of 3 248 patients met our criteria for severe cervical spine injury, and the number of patients was relatively stable over the 4-year period (Table 2). A total of 2 963 patients had one or more CS-fx, and 285 patients had severe non-fracture cervical injuries. The median age was 54 years (range 0-102 years), and 69% of the patients were male. The median ages for women and men were 63 and 52 years old, respectively. A cSCI was reported in 414 of 3 248 (12.7%) patients. Of the patients with CS-fx, 140 of 2 963 (4.7%) were reported to have a cSCI, whereas 274 of 285 (96.0%) of the non-fracture group had cSCIs. The median age of the patients with a cSCI was 48 years (range 0-95 years), and 74% of these patients were male.

**Table 2:** **Patient characteristics**

		**Number of patients**				
		**2009**	**2010**	**2011**	**2012**	**Total**
Age group (years)	0-14	22	24	18	18	82
	15-29	117	143	138	129	527
	30-44	127	140	142	123	532
	45-59	159	205	145	188	697
	60-74	153	166	166	180	665
	75-89	152	120	149	174	595
	≥90	24	36	42	48	150
	Total all ages (male)	754 (518)	834 (570)	800 (565)	860 (585)	3 248 (2 238)
Injury type	Cervical spine fractures	687	754	732	790	2963
	Severe non-fracture cervical spine injuries	67	80	68	70	285
Cervical spinal cord injuries		100	106	110	98	414
Treatment	Open surgery	140	146	160	153	599
	External fixation with halo vest only	12	8	5	4	29
	No open surgery or halo vest	602	680	635	703	2 620

### Incidence of severe traumatic cervical spine injury in the Norwegian population

**Figure 1:**
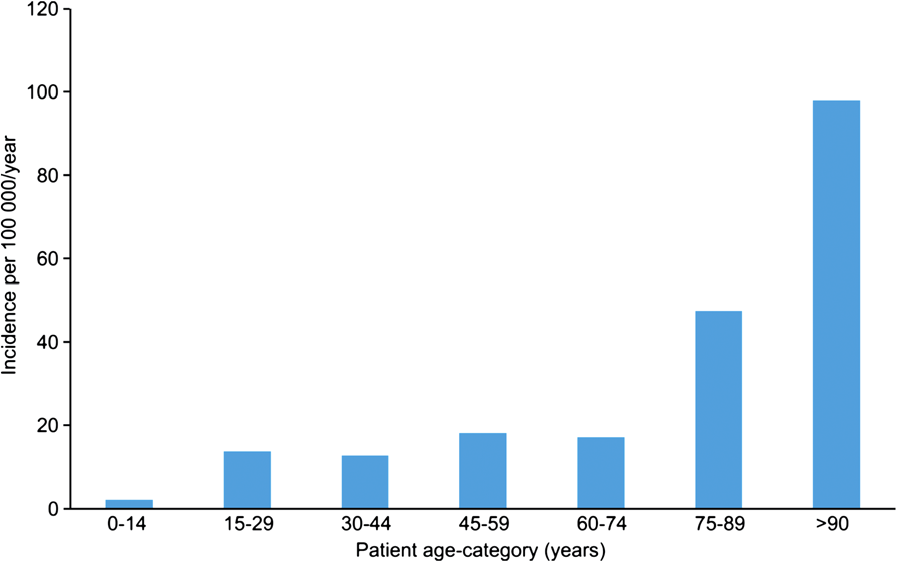
**Age-stratified incidence of traumatic cervical spine injury.**

As of January 1, 2011, the Norwegian population was 4 920,000, which corresponded to an incidence of severe traumatic cervical spine injuries of 16.5/100 000/year (95% CI: 15.4-17.7/100 000/year) in the total population. The incidence of severe cervical spine injury increased significantly with age (p < 0.001) (Figure 1). The incidence of CS-fx was 15.0/100 000/year (95% CI: 14.0-16.2/100 000/year), and the incidence of severe non-fracture cervical spine injury was 1.5/100 000/year (95% CI: 1.3-1.7/100 000/year).

### Open surgical treatment

A total of 599 (18%) patients were treated with open surgery. Of these patients, 570 were treated with open surgical fixation with or without decompression, and 29 were treated with decompression alone. These data yielded an estimated incidence of surgery for acute traumatic cervical spine injury in the Norwegian population of 3.0/100 000/year (95% CI: 2.6-3.6/100 000/year). Of the 414 patients with a cSCI, 115 (28%) underwent an open surgical procedure.

Halo vests are not frequently used in Norway, and only 29 patients with this isolated procedure code were registered during the 4-year period.

### Mortality

Of the 3 248 patients included in this study, 132 died within 1 month of their first admission, and 187 died within 3 months of their first admission. Thus, the 1- and 3-month mortality rates were 4% and 6%, respectively.

- In total, 58% of the patients who died within 3 months of injury were male, and their median age was 84 years (range 8-100 years).
- The risk of death within 3 months of a traumatic cervical spine injury in patients <75 years of age and >75 years of age was 2% and 18%, respectively.
- In the surgery group, the mortality rate was 2% at 1 month and 4% at 3 months.
- In patients with cSCI, the mortality rate was 5% at 1 month and 6% at 3 months.

## Discussion

This is a registry-based study analyzing the epidemiology of severe traumatic cervical spine injuries in a general population. The estimated total incidence of severe traumatic cervical spine injuries in the Norwegian population was 16.5/100 000/year. The incidence of cervical spine fractures and severe non-fracture cervical injuries was 15.0/100 000/year and 1.5/100 000/year, respectively. The incidence of open surgery for severe traumatic cervical spine injuries in the Norwegian population was 3.0/100 000/year.

We selected data from a four-year period, as this was the time-frame we had available with registrations coupled to a personal identification number for each patient. The four years were selected to strengthen the results compared to one-year material, but we had no intention to look for alterations over time, realizing that a four-year period is much too restricted for this purpose.

Our incidence of 15.0/100 000/year for CS-fx in the total Norwegian population was higher than we previously reported. In a previous study, we found that the incidence of CS-fx in the Southeast Norwegian population was 11.8/100 000/year, which we acknowledged as an underestimate [3]. A Canadian study from 1996 reported incidence rates in a general population [2]. In the Canadian study, the incidence of all spinal fractures was 64/100 000/year, and subgrouping the injuries into cervical, thoracic and lumbar fractures was only performed for 45% of the patients, those admitted to hospitals. Based on the data from hospitalized patients, the estimated CS-fx incidence in the general Canadian population was 12/100 000/year.

A Swedish study from 2002 published numbers of Cs-fx from a nationwide population [1]. Based on patient data from 1987 to 1999, the incidence of Cs-fx in the Swedish population declined from 10.5/100 000/year in 1987 to 9.2/100 000/year in 1999. The proportion of CS-fx patients with a concomitant cSCI was 51% (1987) and 36% (1999). The latter finding suggests that a significant proportion of the CS-fx was missed in the Swedish data registry, and that their numbers of patients with CS-fx are underestimates. The lifestyles in Sweden and Norway are in general quite comparable, as with most developed countries.

We assume that the actual incidence of severe traumatic cervical spine injuries is even greater than our reported estimate of 16.5/100 000/year for the following reasons.

1. Data from traumatic cervical spine injuries causing immediate death at the scene or at the time of hospital admission were not available. Previous reports have suggested that 21-24% of victims who die immediately or soon after a traffic accident have a serious injury to the cervical spine [23,24]. Approximately 2 000 people die of traumatic causes in Norway each year, and approximately 200 of these are due to traffic-related accidents. A study on patterns of trauma deaths within a defined region in Western Norway found an incidence of prehospital trauma-related death to be 5.2/100 000/year [25]. Within this prehospital fatality group, 56% of the causes of death were related to the central nervous system, though not further divided into cerebral and medullary injury.
2. Patients with a missed diagnosis of a cervical spine injury are not registered. These individuals include patients who do not receive health-care services after an injury or patients with a missed diagnosis despite assessment.
3. If a patient was repeatedly admitted to hospitals during this 4-year period with two or more separate traumatic cervical spine injuries, the injuries were registered as one event in our data set.
4. Patients with occipital condyle fractures were missed in this type of study. While this injury type is considered a subgroup of CS-fx, the diagnostic code is a skull-base fracture. These injuries could represent as many as 5% of CS-fx cases [3].
5. We have chosen a stringent approach regarding which cervical discoligamentous injuries we have enrolled, as we only included those with a relevant surgical procedure. We believe the coding of S13 to be less reliable than S12. To avoid including a large number of unspecific cervical soft tissue injuries, we accepted this limitation. However, this restricion might have caused us to miss some relevant severe non-fracture cervical spine injuries.

We assume that these “missed cases” represent less than 10% of all severe traumatic cervical spine injuries. Thus, in Norway, the “true incidence” of severe traumatic cervical spine injuries are most likely approximately 18/100 000/year. On the other hand, if patients treated for a cervical spine injury in 2007 or earlier were readmitted during 2009-2012 for the same condition, with the same diagnostic codes, they were falsely registered as new cases. We do not believe there were significant number of these.

We observed a predominance of men in our patient population. This finding is in accordance with previous reports [1-3,6,8-10,14-17,26-29]. The age distribution in our series revealed that the greatest frequency of traumatic cervical spine injuries occurred in individuals between the ages of 45 and 89 years. When adjusted for the age distribution in the Norwegian population, we found the greatest incidence in the oldest age group. This result is consistent with our earlier findings in Southeast Norway. However, the results contradict most earlier findings that traumatic cervical spine injuries were most common in the third decade of life or between 15 and 45 years old [8,15-17,26,30,31]. Some authors also reported a second “peak” at age 65-80, similar to our results [1,14,30,32]. As reported by others, we found a low incidence of cervical spine fractures in children [1,3,4,14,18,32]. The older age among our patients was most likely because our data were from a total general population, whereas most other reports only focused on specific subpopulations.

The prevalence of cSCI among patients with CS-fx has been reported to be 10-51% [1,3,5,8,9,11,16,26,31,32]. In this study, 12.7% of the patients had a diagnostic code for cSCI. Of the patients with CS-fx, only 4.7% were reported to have a cSCI, whereas 96.0% of the non-fracture group had a cSCIs. In fact, the 4.7% prevalence of cSCI in the patients with CS-fx was substantially lower than the 10% prevalence we registered in our previous Southeast Norway population study [3]. In the Southeast Norway study, we registered CS-fx in approximately half of the Norwegian population during an overlapping time period. All of the patients were referred to the only trauma center in the region that performed surgery on traumatic cervical spine injuries, and the data were retrieved using a chart review. Based on these quality-assured data at the single-patient level, it is evident that the NPR under-reports cSCI when there is a concurrent CS-fx. We believe that this issue occurs because clinicians often forget or omit the code (ICD-10) for cSCI when they use fracture as the main code. Therefore, the cSCI numbers presented here were substantially underestimated.

In the Southeast Norway study, we acknowledged that a minor proportion of patients were not referred to the regional main trauma center for consultation/treatment and were therefore missed. This hypothesis is supported by the current NPR numbers. It is reasonable to assume that virtually all patients with a cSCI were referred and that patients with uncomplicated CS-fx without a concurrent cSCI were treated in local hospitals. Under the assumption that the Southeast Norway study provided the most accurate estimation of CS-fx with cSCI and that the current study provides the most accurate estimation of all CS-fx, the true prevalence of cSCI in patients with CS-fx is 7%. From this value, we could estimate the incidence of all cSCIs to be 2.6/100 000/year.

A total of 18% of the patients were treated with open surgery, and accordingly, the estimated incidence of surgery for acute traumatic cervical spine injury in the Norwegian population was 3.0/100 000/year. We have not found any reports with comparable data to evaluate whether this is a high or low number. All of the neurosurgical clinics in Norway use established classification systems for traumatic cervical spine injuries. In addition, the available treatment recommendations and guidelines are well known. In our previous Southeast Norway study, we found that our treatment of subaxial CS-fx matched the treatment recommendations of the Subaxial Injury Classification System (SLIC) [33] in more than 90% of patients. These data indicate that our compliance with the established guidelines was high. However, these registry data were not suitable for more specific analyses of surgical treatment.

Halo vests are not frequently used in Norway, with only 29 patients registered with this isolated procedure code within the four-year period. There are regional differences, as the Central region had by far the largest proportion of patients receiving halo vests.

Less than one-third (28%) of the patients with cSCI were treated with open surgery. This seems like a low rate. As discussed earlier, we have acknowledged that the diagnostic codes for cSCI in patients with concomitant CS-fx are underreported. Therefore, we have reason to believe that a larger proportion of these patients with both CS-fx and cSCI are treated with open surgery. This might lead to a corresponding underestimation error in the surgery rates for patients with cSCI.

The ICD-10 diagnostic codes for cSCI (S14.0 and S14.1) specify neither severity nor type/syndrome of cSCI. It is our belief that a significant portion of non-fracture cSCIs is central cord syndrome in elderly patients who sustain trauma to a predisposed spondylotic cervical spine.

The overall 1- and 3-month mortality rates in this study were 4% and 6%, respectively, with lower figures for surgically treated patients (2% and 4%, respectively). The overall mortality was low in patients <75 years old (2%) but high in patients >75 years old (18%). The mortality causes are not included in these data, and we have no basis for assuming that the cervical injury was the direct cause of death in the majority of the patients. From our clinical knowledge we are aware that a proportion of these patients have suffered multi-organ trauma. The high mortality rate in the elderly population may be partly due to pre-existing co-morbidities. In our previously published material based on the Southeast Norway population, the total mortality rates were slightly higher (7% and 9%, respectively). The figures for surgically treated patients were almost identical (2% and 3%, respectively) [3]. In the Swedish national study, the case fatality rate in patients with CS-fx declined throughout the study period from 9 to 4% [1]. According to the mortality rates that have been reported in different subgroups, the mortality in our population is within the lower range [16,17,31,34-36].

This study provides an example of the advantages of using administrative health registries in epidemiological studies. Nationwide and mandatory data reporting prevents selection bias and loss to follow-up, and it provides an excellent framework for surveillance and research. Information on deaths outside hospitals is also readily available through routine registry linkage to the General Register Office.

Having prospective, chart-based clinical data from the same patient group from the largest of the four health regions over an overlapping time period provided us with an excellent opportunity to compare and consider the quality of these registry-based data. For instance, we found full compliance in the number of surgically treated patients in the registry data compared to the chart-based regional data (data not shown). In contrast, the chart-based regional data indicated that the diagnostic coding for cSCI was incomplete in the registry data, resulting in the under-reporting of the prevalence of cSCI in patients with CS-fx. Thus, to ensure the greatest level of accuracy, the estimate must be calculated based on a synthesis of registry data and chart-based regional data.

The main limitations of the study were related to the study design, which involved the retrieval of information from a registry database. Accordingly, we did not have access to outcome data other than deaths. Additionally, direct patient information was unavailable, so we could not differentiate between injury mechanisms and types of injury by classification. Furthermore, we could not control for confounding variables, such as predisposing individual factors and morbidity.

The diagnostic (ICD-10) and procedure codes (NSCP) possess limitations. Although ICD-10 is more detailed in regard to discoligamentous injuries than the previous ICD-9, the codes are still limited in regard to details in terms of stability and severity, as mentioned when discussing cSCI. And the greatest challenge; there are personal and regional variations in use of the codes, regarding interpretation, accuracy and completeness. The NSCP codes holds many of the same limitations.

Another limitation of the study was the relatively recent establishment of the NPR as a registry with individual-level data and the lack of data validation. Validation studies would strengthen the research value of NPR data. However, we observed good concordance between the diagnosis and procedure codes and greater consistency in yearly distributions, which we interpreted as a good indicator of high data validity. Furthermore, the personal identification reporting was nearly complete (98%), which allowed for confident monitoring of the same patients treated in different hospitals on repeated admissions.

## Conclusions

- The incidence of severe traumatic cervical spine injury in the general Norwegian population is estimated at 16.5/100 000/year. The incidence of traumatic cervical spine fractures is estimated at 15.0/100 000/year and the incidence of severe non-fracture cervical spine injury at 1.5/100 000/year.
- The incidence of open surgery for acute traumatic cervical spine injury is estimated at 3.0/100 000/year.
- The 1- and 3-month mortality rates were 4% and 6%, respectively. The risk of death within 3 months after a traumatic cervical spine injury was 2% in patients <75 years old and 18% in patients >75 years old.
- Cervical spine injuries occurred most frequently in the 45- to 89-year age group. When adjusted for age distribution, we found the highest incidence in the oldest age group.
